# Bat Flight and Zoonotic Viruses

**DOI:** 10.3201/eid2005.130539

**Published:** 2014-05

**Authors:** Thomas J. O’Shea, Paul M. Cryan, Andrew A. Cunningham, Anthony R. Fooks, David T.S. Hayman, Angela D. Luis, Alison J. Peel, Raina K. Plowright, James L.N. Wood

**Affiliations:** US Geological Survey, Fort Collins, Colorado, USA (T.J. O’Shea, P.M. Cryan); Zoological Society of London, London, UK (A.A. Cunningham);; Animal Health and Veterinary Laboratories Agency—Weybridge, Weybridge, UK (A.R. Fooks);; National Consortium for Zoonosis Research, South Wirral, UK (A.R. Fooks);; Colorado State University, Fort Collins, Colorado, USA (D.T.S. Hayman, A.D. Luis);; University of Florida, Gainesville, Florida, USA (D.T.S. Hayman);; Fogarty International Center of the National Institutes of Health, Bethesda, Maryland, USA (A.D. Luis);; University of Cambridge, Cambridge, UK (A.J. Peel, J.L.N. Wood);; Griffith University, Brisbane, Queensland, Australia (A.J. Peel);; Pennsylvania State University, University Park, Pennsylvania, USA (R.K. Plowright)

**Keywords:** bats, body temperature, Chiroptera, emerging zoonotic viruses, fever, flight, metabolic rate, mammals

## Abstract

Bats are sources of high viral diversity and high-profile zoonotic viruses worldwide. Although apparently not pathogenic in their reservoir hosts, some viruses from bats severely affect other mammals, including humans. Examples include severe acute respiratory syndrome coronaviruses, Ebola and Marburg viruses, and Nipah and Hendra viruses. Factors underlying high viral diversity in bats are the subject of speculation. We hypothesize that flight, a factor common to all bats but to no other mammals, provides an intensive selective force for coexistence with viral parasites through a daily cycle that elevates metabolism and body temperature analogous to the febrile response in other mammals. On an evolutionary scale, this host–virus interaction might have resulted in the large diversity of zoonotic viruses in bats, possibly through bat viruses adapting to be more tolerant of the fever response and less virulent to their natural hosts.

## Abstract 2

High metabolism and body temperatures of flying bats might enable them to host many viruses.

Bats are a major source of zoonotic viruses worldwide (*1*–*3*). Molecular studies have demonstrated that bats are natural host reservoirs for several recently emerged high-profile zoonotic viruses, including sudden acute respiratory syndrome–like coronaviruses (*4*); Ebola and Marburg hemorrhagic fever filoviruses (*5**,**6*); rabies and rabies-related lyssaviruses; and many paramyxoviruses, including rubulaviruses and Nipah and Hendra viruses (*7**–**9*). Identification of a diverse range of bat paramyxoviruses, including those conspecific with human mumps virus, and phylogenetic reconstruction of host associations suggests numerous host switches of paramyxoviruses from bats to other mammals and birds (*9*). Bats infected with these viruses seem to show no overt signs of disease (*10*,*11*) and, in some cases, appear to be persistently infected (*12*). In the aggregate, zoonotic viruses in >15 virus families have been identified in at least 200 species in 12 bat families around the world (*3*,*9*,*11*). In a recent comparative analysis, Luis et al. (*3*) showed bats to be more likely to be infected with more zoonotic viruses per host species than were rodents, thus adding weight to the suggestion that bats might in some way be unique as sources of emerging zoonoses. In areas where these viruses have been studied, some viral groups (e.g., coronaviruses, astroviruses, paramyxoviruses) have much higher viral diversity and prevalence in bats than in sympatric species of rodents (*9*,*11*).

Although bats serve as reservoir hosts with great viral diversity, little evidence exists for corresponding death or illness of bats from viruses (other than lyssaviruses) that have spread into humans and domesticated mammals with high virulence (*9*,*10*). This statement also can be true for some viruses from other groups of mammals that serve as reservoirs of viruses, particularly rodents. However, low virulence coupled with high diversity has led to the suggestion that bats might have evolved mechanisms to control viral replication more effectively than have most other mammals (*10*,*13*) and that some attribute common to all bats (a very widely distributed and diverse group) also might explain the apparent low virulence of viral infections in these animals (*1*,*2*). We hypothesize that the increased metabolism and higher body temperatures of bats during flight might serve as an evolutionary adjuvant to their immune systems, providing a powerful selective force against virulence and promoting the diversity of viruses that infect bat populations. Perhaps counter-intuitively, this would enable bats to tolerate a greater diversity of viruses that have a high potential for virulence when transmitted to other mammals. The hypothesis also might help explain why co-evolved bat viruses cause high pathogenicity when they spill over into other mammals because the bat-derived viruses might survive well under both febrile and cooler conditions.

## Are Bats Special As Viral Hosts?

Three recent reports provide especially noteworthy background for this perspective. Luis et al. (*3*) performed a comparative analysis of viruses in bats and rodents (the most speciose group of mammals) and associated ecologic factors. The analysis indicated that bats are indeed special in hosting more viruses per species than rodents, despite twice as many rodent species in the world, and that certain ecologic factors are associated with the hosting of more viruses by bats (*3*). Through an extensive genomic analysis of 2 divergent species of bats, Zhang et al. (*13*) found that flight evolved in tandem with concomitant genetic changes to their innate immune systems. These changes were consistent with the need for DNA damage repair because of high metabolic rates during flight (*13*). Baker et al. (*10*) reviewed antiviral immune responses in bats and suggested the possibility that bats might be able to control viral replication through innate immunity. They summarized research indicating that bats have immune elements found in most other mammals, including pattern recognition receptors and multiple interferons, and show complement activity (*10*). Many standard elements of the adaptive immune system also have been described in bats, including multiple immunoglobulins, antibody responses, interleukins and other cytokines, and cell-mediated T-cell responses (although investigations of the latter have been limited [*10*]). However, genes that code for some immune elements are notably lacking in bats and indicate permanent change to the bat immune system (*13*).

## Fever, Flight, and Metabolic Rate

Fever in mammals is accompanied by an initially high metabolic rate associated with beneficial immune responses (*14*,*15*). During flight, bats exhibit a high increase in metabolic rate over the resting metabolic rate of normeothermic, otherwise active bats. This increase is estimated to be 15–16-fold (*16*), in comparison with the 7-fold increase in metabolic rates of rodents running to exhaustion (*17*) or the 2-fold increase in metabolic rates of most flying birds (*18*). Strains of laboratory mice that are inbred for higher metabolic rates show stronger immune responses to immune challenge (keyhole limpet hemocyanin antigen) with stronger antigen-specific IgM production than strains bred for lower metabolic rates; leukocyte counts and mass of lymphatic organs that are the sources of immune cells involved in antigen recognition and elimination also are elevated in the strains with higher metabolic rates (*19*). The metabolic cost of raising an immune response to experimental stimulation typically results in a general increase of ≈10%–30% of resting metabolic rates in a variety of nonvolant small mammals (*20*,*21*). When a bat is confronted by a viral antigen, the proportional increase in metabolism for raising an immune response may be trivial compared with the very large increase in the metabolic costs of flight (the proportional increase for flight may even be greater, given the wider metabolic scope of many species of bats that undergo shallow daily torpor). Thus, we hypothesize that the higher metabolic rates during flight in bats may enhance, facilitate, or perhaps subsidize any inherent cost of raising metabolism to activate an immune response. The daily cyclical nature of raised metabolism during flight also might enable some viruses to persist within the bats and perhaps become resistant to this part of the innate immune response.

## Bat Flight and Elevated Body Temperatures

Canale and Henry (*22*) stated, “The heat of fever forestalls pathogen replication and increases the efficiency of the immune responses. Such body warming is associated with shortened disease duration and improved survival in most animals.” Although fever has been associated with improved recovery, very little is known about mechanisms, including whether the impact involves thresholds or average rates of immune response. During fever, mammalian core body temperatures can vary but typically are 38°C–41°C (*14*). The high metabolic demands of bat flight result in core body temperatures that commonly reach the ranges of core temperatures typical of fever. High body temperatures during flight have been demonstrated in multiple species of bats within several families (Table 1), and such high body temperature ranges increase the rate of multiple immune responses in mammals, including components of the innate and adaptive immune systems (Table 2). Daily high body temperatures thus might arm bats against some pathogens during the early stages of infection. An exception to the daily high body temperatures during flight occurs during hibernation in temperate zones: although bats rouse from hibernation multiple times each winter (*35*), replication of most mammalian adapted pathogens is expected to be markedly reduced by the lower core body temperatures of hibernation.

**Table 1:** Examples of elevated core body temperature in flying bats*

Bat species (family)	Core temperature during flight, °C	Source
*Eidolon helvum* (Pteropidae)	36.9–40.8	(*23*)
*Hypsignathus monstrosus* (Pteropidae)	37.2–40.0	(*23*)
*Rousettus aegyptiacus* (Pteropidae)	38.2–41.2	(*23*)
*Rhinolophus ferrumequinum* (Rhinolophidae)	41†	(*24*)
*Miniopterus* sp. (Miniopteridae)	41.1 ± 0.45	(*25*)
*Phyllostomus hastatus* (Phyllostomidae)	41.2–42.1	(*17*)
*Carollia perspicillata* (Phyllostomidae)	40.2 ± 0.8	(*26*)
*Artibeus lituratus* (Phyllostomidae)	c. 41.2 ± 1	(*27*)
*Sturnira lilium* (Phyllostomidae)	c. 40.5 ± 0.3	(*27*)
*Noctilio albiventris* (Noctilionidae)	35.5–40.6	(*28*)
*Myotis yumanensis* (Vespertilionidae)	40.0–40.8	(*29*)
*Eptesicus fuscus* (Vespertilionidae)	41.3 ± 2.1†, 37–39.5‡	(*30*)
*Mops condylurus* (Molossidae)	40.5 ± 1.1	(*31*)
*Tadarida brasiliensis* (Molossidae)	35–42	(*32*)
*Eumops perotis* (Molossidae)	37.8–39.3	(*33*)
*Myotis volans* (Vespertilionidae)	37.4	(*34*)
*Myotis evotis* (Vespertilionidae)	38.3	(*34*)
*Myotis californicus* (Vespertilionidae)	38.4	(*34*)
*Parastrellus Hesperus* (Vespertilionidae)	38.8	(*34*)
*Eptesicus fuscus* (Vespertilionidae)	41.0	(*34*)
*Lasiurus cinereus* (Vespertilionidae)	40.2	(*34*)
*Antrozous pallidus* (Vespertilionidae)	40.6	(*34*)
*Tadarida brasiliensis* (Molossidae)	38.0	(*34*)

*Data available from original sources are given as ranges or means ±1 SD. †Skin temperature. ‡Body temperature.

**Table 2:** Favorable innate and adaptive immune responses associated with the high body temperature of fever in mammals*

Enhanced neutrophil and monocyte motility and emigration
Enhanced phagocytosis and pinocytosis
Increased oxygen radical production by phagocytes
Increased interferon production
Increased antiviral, antitumor, or antiproliferative, and natural killer cell stimulating activities of interferon
Potentiated interferon-induced anti-anaphylaxis (anergy)
Enhanced natural killer complement activation
Enhanced expression of Fc receptors
Increased T-helper cell activation, expression, recruitment, and cytotoxic activity
Blocked T-suppressor cell activity
Increased antibody production
Enhanced tumor necrosis factor-α
Increased T-cell proliferative response to nonspecific mitogens, interleukin-1 and −2, and allogeneic lymphocytes
Increased killing of intracellular bacteria
Increased bactericidal effect of antimicrobial agents
Induced cytoprotective heat-shock proteins in host cells
Induced pathogen heat-shock proteins, which activate host defenses
Induced cytoprotective heat-shock proteins in host cells

*See reviews in (*14**,**15*).

## A Speculative Hypothesis

If the elevated metabolic rates and body temperatures accompanying flight facilitate activation of the immune system of bats on a daily cycle, then flight could be the ultimate explanatory variable for the evolution of viral infections without overt signs of illness in bats. Zhang et al. (*13*) showed that the evolution of flight in bats has been accompanied by genetic changes to their immune systems to accommodate high metabolic rates. Theoretical models of the evolution of parasite virulence show that intermediate levels of virulence are a typical result of the trade-off between the opposing selective forces of host death and parasite transmission (*36*). However, heightened host adaptive immune responses that might be facilitated in bats during flight also can result in harmful immunopathologic changes and disease. (A nonviral example of such harmful immunopathology in bats seems to occur during infection by the fungal pathogen causing white-nose syndrome (*Pseudogymnoascus destructans*) when hibernating bats resume flight [*37*]). In systems in which disease organisms cause major immunopathologic changes, theoretical analyses suggest that natural selection can favor decreased virulence and incomplete clearance of parasites (*36*,*38*). Through heightened immunopathologic responses, flight might have been a potent selective factor for the reduced virulence to the natural hosts seen in the pool of emerging viruses recently discovered in bats. It also is notable that there are few reports of mass deaths from diseases in bats (except for the novel white-nose syndrome fungus), despite reports of bat die-offs due largely to other causes that have appeared in the literature over the years (e.g., review in *39*). The evolution of flight in conjunction with the bat’s speculated heightened potential for immune vigilance might have predisposed bats to be reservoir hosts to a preponderance of viruses that now lack major effects on bats as the natural host populations but that can emerge into populations of humans and domesticated mammals with greater virulence.

Consideration of the role of torpor is also germane to our hypothesis. As noted by Luis et al. (*3*), “more research is needed to determine the relationship between torpor, host competence as related to within-host viral persistence and population viral perpetuation processes.” Viral replication is dampened under the cooler host body temperatures that prevail during prolonged torpor, and hypothermia has been considered to be a host strategy that is adaptive against pathogens (*40*). Luis et al. (*3*) hypothesized that the negative correlation identified between the use of torpor and zoonotic viral richness may be due to lower contact rates, yet in bats it is also consistent with longer periods with no or lower flight activity and, consequently, lower frequency of the hypothesized heightened vigilance against invading viruses during the course of host–parasite co-evolution. On the other hand, reduced immune system activity during torpor may enable cold-adapted pathogens to persist (*40*), as in the case of the novel fungal pathogen causing white-nose syndrome. As a group, bats show a wide range of adaptations involving torpor that varies with latitude and phylogeny, ranging from prolonged deep winter hibernation through shallow daily torpor to year-round homeothermy. Viruses that have co-evolved with bats under these conditions might have properties that can favor survival under a wider scope of temperatures, possibly facilitating spillover to novel hosts.

## Testing the Hypothesis

In their review of bat immunology and viral diversity, Wang et al. (*11*) posed the question “Flight capability, longevity and innate immunity—are they linked?” and noted that “data in this field are so limited that it is … important to provoke original, speculative or even controversial ideas or theories in this important field of research.” Our “flight-as-fever” hypothesis suggests 1 mechanism unique to bats that might be key in the flight capability–innate immunity linkage question raised by Wang et al. (*11*). Researchers interested in testing this hypothesis will find it challenging and demanding of creativity. Unfortunately, no prior studies have investigated the effect of the high body temperatures and metabolic rates associated with flight on host–virus interactions in bats.

However, we suggest that a variety of in vivo, in vitro, and in silico approaches be considered. For example, in vivo approaches could rely on the experimental techniques that were used decades ago in the pioneering studies of bat flight physiology (*16*,*17*). As in these prior physiologic studies, captive bats can be trained to fly in wind tunnels. Experiments could be designed to determine whether trained bats allowed to fly show heightened immune responses compared with when they were not flying. These experiments could begin with determining immune responses after exposure to harmless antigens and then progress to experiments involving exposure to viruses. In vitro studies could determine the comparative susceptibility of bat viruses and nonbat viruses grown in culture to altered and variable thermal regimes typical of the body temperatures of bats during flight, as well as bats in torpor. (In this regard, we note that most bat viruses that have been identified by using genetic techniques have not been isolated, described morphologically, or grown in culture; additional research using techniques of classical virology are certainly needed to improve the understanding of bat virology). Finally, in silico techniques of modeling and simulation would be helpful in understanding the likely co-evolution of bat viruses and their hosts when subject to daily fever-like thermal and metabolic regimes. Regimes of such frequency might accelerate the pace of co-evolution in the otherwise slowly evolving hosts, and favor the development of low pathogenicity in their much more rapidly evolving viruses.
